# Carbapenemase-producing Enterobacterales strains causing infections in companion animals—Portugal

**DOI:** 10.1128/spectrum.03416-23

**Published:** 2024-03-06

**Authors:** Joana Moreira da Silva, Juliana Menezes, Laura Fernandes, Sofia Santos Costa, Andreia Amaral, Constança Pomba

**Affiliations:** 1 CIISA—Centre for Interdisciplinary Research in Animal Health, Faculty of Veterinary Medicine, University of Lisbon, Lisbon, Portugal; 2 AL4AnimalS—Associate Laboratory for Animal and Veterinary Sciences, Vila Real, Portugal; 3 Global Health and Tropical Medicine, GHTM, Instituto de Higiene e Medicina Tropical, IHMT, Universidade Nova de Lisboa, UNL, Lisbon, Portugal; 4 Genevet, Veterinary Molecular Diagnostic Laboratory, Carnaxide, Portugal; Universidade de Sao Paulo, São Paulo, Brazil

**Keywords:** veterinary, diagnostics, KPC-3, *Klebsiella pneumoniae*, OXA-48, *E. coli*, OXA-181

## Abstract

An increase in *Klebsiella pneumoniae* carbapenem-resistant human nosocomial strains is occurring in Europe, namely with the *bla*_OXA-48-like_ and *bla*_KPC-like_ genes. We determined the prevalence of carbapenemase-producing Enterobacterales clinical strains in companion animals in Portugal and characterized their mobile genetic elements. Susceptibility data of a consecutive collection of 977 Enterobacterales clinical strains from a Portuguese private veterinary diagnostic laboratory were evaluated (January–December 2020). Additional phenotypical and genotypical assays were performed in a subset of 261 strains with a resistant phenotype. Whole-genome sequencing was performed for carbapenemase-producing strains. The frequency of carbapenemase-producing Enterobacterales clinical strains in companion animals in Portugal was 0.51% (*n* = 5/977). Thus, five strains were characterized: (i) one OXA-181-producing *K. pneumoniae* ST273, (ii) two KPC-3-producing *K. pneumoniae* ST147; (iii) one KPC-3-producing *K. pneumoniae* ST392; and (iv) one OXA-48-producing *E. coli* ST127. The *bla*_KPC-3_ gene was located on transposon Tn*4401d* on IncFIA type plasmid for the *K. pneumoniae* ST147 strains and on a IncN-type plasmid for the *K. pneumoniae* ST392 strain, while *bla*_OXA-181_ gene was located on an IncX3 plasmid. All *de novo* assembled plasmids and plasmid-encoded transposons harboring carbapenemase genes were homologous to those previously described in the human healthcare. No plasmid replicons were detected on the OXA-48-producing *E. coli* ST127. The dissemination of carbapenem resistance is occurring horizontally *via* plasmid spreading from the human high burden carbapenem resistance setting to the companion animal sector. Furthermore, companion animals may act as reservoirs of carbapenem resistance. Implementation of carbapenemase detection methods in routine clinical veterinary microbiology is urgently needed.

**IMPORTANCE:**

This is the first study on the prevalence of carbapenemase-producing Enterobacterales (CPE) clinical strains from companion animals in Portugal. Despite the generally low prevalence of CPE in companion animals, it is imperative for veterinary diagnostic laboratories to employ diagnostic methods for carbapenemase detection. The resemblance found in the mobile genetic elements transporting carbapenemase genes between veterinary medicine and human medicine implies a potential circulation within a One Health framework.

## INTRODUCTION

Resistance to carbapenems by Enterobacterales poses a threat to healthcare systems worldwide. Such infections are associated with high mortality, with limited options for treatment available (1). The 2021 European Centre for Disease Prevention and Control (ECDC) report demonstrates an increase in carbapenem-resistant *Escherichia coli* and *Klebsiella pneumoniae* strains. While the prevalence of *E. coli* strains resistant to carbapenems remains low, a quarter of European countries report a prevalence above 10% in *K. pneumoniae* carbapenem-resistant strains (2).

The most predominant genes across Europe are the *bla*_OXA-48-like_ and *bla*_KPC-like_ associated with *K. pneumoniae* strains (3). Similarly, in Portugal, a rise in the number of studies reporting *K. pneumoniae* strains resistant to carbapenems in the healthcare setting is observed, with *bla*_OXA-181_ and *bla*_KPC-3_ being the primary genes detected (4, 5).

In 2020, The European Medicines Agency (EMA) performed an update on the categorization of antibiotics available for use in Veterinary medicine which impact public health, where carbapenems are classified as category A (“Avoid use”) (6). Yet, carbapenem-resistant bacteria have been described either as causative agents of infection or commensal in dogs and cats across the world (7–10). Regardless of the type of studies, most reported bacteria are identified as OXA-48-like-producing Enterobacterales (7–9). In Portugal, three reports of carbapenem-resistant bacteria in companion animals have, so far, been described: (i) an OXA-23-producing *Acinetobacter baumannii* causing a urinary tract infection in a cat (11); (ii) an OXA-181-producing *E. coli* was found as a commensal strain on a dog (12); and (iii) a KPC-3-producing *K. pneumoniae* causing an upper respiratory tract infection in a dog (13), which was previously characterized in-depth by the authors of this study. Thus, a comprehensive screening for the prevalence of carbapenem-resistant bacteria in companion animals is lacking.

The purpose of this retrospective study is to determine the frequency of carbapenem-resistant bacteria causing infection in companion animals and to evaluate the possible transmission of mobile genetic elements carrying carbapenemase genes within the One Health context.

In partnership with a veterinary diagnostic laboratory, a consecutive collection of Enterobacterales strains collected during 2020 was evaluated and whole-genome sequencing (WGS) was used to further characterize carbapenemase-producing strains.

## MATERIALS AND METHODS

### Bacterial strains

Samples for microbiology analysis were sent from different veterinary practices in Portugal to the Genevet Molecular Diagnostic Laboratory. Enterobacterales clinical strains (*E. coli*, *Klebsiella* spp., *Enterobacter* spp., *Citrobacter* spp., *Serratia* spp., and *Proteus* spp.) were identified using MacConkey and chromogenic agar UriSelect™ (Bio-Rad, US). Antibiogram results for a consecutive collection of 977 Enterobacterales clinical strains of 2020 were interpreted according to EUCAST 2023 and Veterinary CLSI breakpoint guidelines (14, 15). Minimum inhibitory concentrations (MICs) were determined using MicroSan NEG44 plates (Beckman Coulter, US) when requested by clinicians. Pure bacterial strains were stored in Brain Heart Infusion liquid media with 20% glycerol at −20°C.

### Enterobacterales strain collection

Within the initial collection, 21 strains were unaccounted for or did not grow when defrosted, thus being excluded from further analyses. Four groups were observed: (i) clinical strains with an ESBL phenotype (*n* = 204); (ii) clinical strains with a possible OXA-48-like phenotype (resistance toward amoxicillin in combination with clavulanic acid while susceptible to cefoxitin and cefotaxime) (*n* = 34) (16); (iii) clinical strains with a multidrug-resistant (MDR) profile as per criteria established by Magiorakos et al., 2012 (17) (resistance to enrofloxacin, gentamicin, tetracycline, and trimethoprim/sulfamethoxazole) (17) (*n* = 23); and (iv) susceptible clinical strains (susceptibility to ampicillin/amoxicillin, amoxicillin in association with clavulanic acid, cephalothin, cefotaxime, ceftazidime, cefoxitin, trimethoprim/sulfamethoxazole, gentamicin, tetracycline, enrofloxacin, and amikacin) (*n* = 692). Due to the observed resistant phenotype, only study groups i, ii, and iii were included. This inclusion criteria resulted in a subset of 261 strains that were additionally phenotypically and genotypically evaluated.

#### Phenotypic screening

Additional phenotypic antimicrobial susceptibility testing for the Enterobacterales strains was carried out against meropenem (10 µg), imipenem (10 µg), temocillin (TMC, 30 µg), and MAST CAT-ID discs (Mast Group, United Kingdom). KPC-3-producing strains were also tested against Ceftazidime-Avibactam (CAZ, 14 µg) by disc diffusion method. Results were interpreted in accordance with EUCAST breakpoint guidelines 2023 (14). TMC discs and MAST CAT-ID discs were used as previously described in Moreira da Silva et al*.,* 2022 (8). For all carbapenemase-producing strains, MIC panel testing was performed and interpreted according to Veterinary CLSI breakpoints or EUCAST breakpoints (14, 15).

#### Genotypic screening via PCR assays and sequencing

DNA was extracted from pure cultures using a boiling extraction method, and a series of multiplex PCRs were performed, as previously described for the detection of extended-spectrum β-lactamase, plasmid-encoded AmpC β-lactamase and carbapenemase genes (18). Sanger sequencing was performed to identify the amplified β-lactamase and carbapenemase genes. Confirmation of species identification was performed by sequencing 16S rRNA as previously described (19).

#### WGS analysis

Strains harboring carbapenemase genes were selected for whole-genome sequencing. Whole DNA was extracted from RNase-treated lysates *via* NZY Tissue gDNA Isolation kit (NZYTech, Lisbon, Portugal). All libraries for WGS were prepared using a TruSeq DNA PCR-Free preparation kit (Illumina, San Diego, California, USA). DNA sequencing was performed using the Illumina NovaSeq platform with 2 × 150 bp paired-end reads. The quality of the resulting raw reads was evaluated using FastQC v0.11.5 (https://www.bioinformatics.babraham.ac.uk/projects/fastqc/), read quality filtering was performed using PRINSEQ v0.20.4 (20) using the following criteria, mean base quality score ≥20 and minimum read length of 90 nt. *De novo* genomes were assembled using SPAdes v3.14.1 (21), following two rounds of polishing using Pilon v1.24 (22). Finally, genome annotation was performed using Prokka v1.14.6 (23). Resfinder 4.1 was used to screen the novel generated assemblies for antimicrobial resistance genes, while Mobile Element Finder v1.0.3 and PlasmidFinder v2.1 were used to locate the antimicrobial resistance genes in the generated assemblies and plasmid’s incompatibility group, respectively. MLST 2.0 and plasmid MLST 2.0 (pMLST) were also performed as typing tools for the *de novo* generated genomes and plasmids identified, respectively. In addition, for the *E. coli* isolate, the tool VirulenceFinder 2.0 was also used to identify the existing virulence factors. All tools are available at the Centre of Genomic Epidemiology (https://www.genomicepidemiology.org/). Pathogenwatch online platform was used to search for outer-membrane protein alteration on the *K. pneumoniae* strains (24).

#### Phylogenetic analysis

Parsnp v1.2 was used to create a multiple sequence alignment (MSA) of the generated assemblies plus the reference genome *K. pneumoniae* ST258 NJST258_2 (GCF_000597905.1) and publicly available *K. pneumoniae* sequences Clonal Group (CG) 147 isolated in Europe of human origin (Table S2: Data used to generate *Klebsiella pneumoniae* Clonal Group 147 phylogenetic tree) (25). The obtained MSA was used as an input by Gubbins to generate a phylogeny of corrected for recombination events with bootstrapping (100 replicates) (26). The obtained tree and MSA corrected for recombination were submitted to Raxml-NG to infer a phylogeny with bootstrapping support (27). iTOL software was used to visualize the obtained tree (28).

#### Plasmid comparison

Using the BRIG analysis tool (29), obtained contigs that contained antimicrobial resistance genes were aligned to a reference plasmid on the NCBI database (Table S3: Accession numbers and relevant information on plasmids used for the study’s contigs circularization).

## RESULTS

### Antimicrobial susceptibility of the initial collection by the veterinary microbiology diagnostic laboratory

The first approach to search for possible carbapenemase-producing Enterobacterales (CPE) strains was to assess the susceptibility patterns in a collection of consecutive samples from 2020, which resulted in 977 strains. The phenotypic evaluation of all these strains is described in Table S1. It was possible to observe resistance to critically important antimicrobials for human medicine, according to the WHO classification (30), particularly the high prevalence of resistance to third-generation cephalosporins (204 clinical strains with an ESBL phenotype, 20.8%), followed by resistance to fluoroquinolones. Of importance, resistance to trimethoprim/sulfamethoxazole (classified as category D by EMA) (6) was also high (Table S1).

Some strains (*n* = 62, 6.4%) had extended susceptibility information on their MIC including the evaluation of carbapenems. Results are present in Table S4 (Table S4: Routine carbapenem minimal inhibitory concentration results for clinical strains; *n* = 62). Only one possible CPE was identified—a *Klebsiella* spp. strain resistant to ertapenem (MIC >1 mg/L).

### Antimicrobial susceptibility and molecular carbapenemase confirmatory testing

Resistant clinical strains according to origin and type of infection are reported in Table S5. The most prevalent β-lactamase gene found was the *bla*_TEM-1_ gene (*n* = 152; 58.2%). Within the ESBL group (*n* = 204), 23.5% of *K. pneumoniae* and 11.3% of *E. coli* strains harbored the *bla*_CTX-M-15_ genes. All found β-lactamase genes are reported in Table S6 (Table S6: β-lactamase genes detected in clinical strains from companion animals; *n* = 261).

Results from the phenotypic screening for carbapenem resistance are depicted in Table 1. Genotypic confirmatory assays showed that within *K. pneumoniae* strains resistant to carbapenems, three were positive for the *bla*_KPC-3_ gene (VG313, VG314, and VG380 strains). The same strains exhibited growth up to the MAST CAT-ID disc. Furthermore, among the TMC-resistant strains, one was an OXA-181-producing *K. pneumoniae* strain (VG117 strain) and the other was an OXA-48-producing *E. coli* strain (VG204 strain). As expected, due to the ertapenem resistance, the OXA-181-producing *K. pneumoniae* strain had been previously identified at the veterinary diagnostic laboratory as a possible carbapenemase-producing strain.

**TABLE 1:** Phenotypic carbapenem screening of Enterobacterales clinical strains from study groups i to iii (*n* = 261)

Bacterial species	Resistance to Meropenem^*a*^ (10 µg)	Resistance to Imipenem^*a*^ (10 µg)	Resistance to Temocillin^*b*^ (30 µg)	MAST CAT-ID^*b*^
*Escherichia coli*	1	1	4	1
*Klebsiella pneumoniae*	5	6	2	10
*Enterobacter cloacae* complex	0	0	2	2
Bacterial species other than *Proteus* spp.	2	2	0	0

^*a*^ Carbapenem resistance was determined in accordance with EUCAST breakpoint guidelines 2023 (14). Imipenem resistance R < 19 mm; Meropenem resistance <16 mm.

^*b*^ Resistance was defined when the bacterial growth was in contact with the disc.

Both VG204 and VG313 strains were the causative agents of urinary tract infection in a cat and a dog, respectively. VG314 strain was isolated from a dog suffering from skin and soft tissue infection, while the VG117 strain was isolated from an oesophagostomy tube infection site in a cat. VG380 strain was the causative agent of an upper respiratory tract infection in a dog.

In this study, we have found that the frequency of carbapenemase-producing Enterobacterales clinical strains in companion animals in Portugal was 0.51% (*n* = 5/977). These strains were further selected for WGS analysis.

### Carbapenemase-producing-Enterobacterales characterization using whole-genome sequencing

Properties of the obtained assemblies are described in Table S7 (Table S7: Assemblies properties following WGS analysis).

OXA-181-producing *K. pneumoniae* strain belonged to the sequence type 273, while two of the KPC-3-producing *K. pneumoniae* strains belonged to ST147 (*K. pneumoniae* VG313 and VG314 strains) and the KPC-3-producing *K. pneumoniae* VG380 strain belonged to ST392. Both ST273 and ST392 are a single locus variant of ST147 (31); thus, all strains belong to Clonal Group (CG) 147, sublineage 147 (32). The obtained *K. pneumoniae* assemblies were investigated for the presence of resistance genes, with results showing that all harbored a set of resistance genes, which contributes to their MDR profiles, including *bla*_CTX-M-15_ and *oqxB/A* genes (Table 2; Table S8: antimicrobial MICs and resistance genes identified during WGS analysis on carbapenemase-producing *K. pneumoniae* strains). All KPC-3-positive strains were susceptible to the combination disc of ceftazidime-avibactam. Both KPC-3-producing *K. pneumoniae* ST147 strains carried the *ybt16* gene on the integrative conjugative element *ICEKp12*, while the *K. pneumoniae* ST273 strain carried the *ybt9* gene on the *ICEKp3* element (Table 2).

**TABLE 2:** Carbapenemase resistance profile, MLST, resistance genes, virulence determinants, and plasmid replicons for carbapenemase-producing Enterobacterales strains

Strain	Month of Isolation	Location of clinic	Carbapenem resistance^*a*^	MLST	Resistance genes	Mutations	Plasmid replicons	Virulence factors^*b*^	Yersiniabactin
OXA-181-producing *K. pneumoniae* (VG117)	April	Lisbon	ERT	273	*aac(6’)-lb-cr,bla*_SHV-1;_ *, bla*_CTX-M-15_ *,bla*_OXA-181,_ *oqxB/A, qnrS1, tetD, sul1,dfrA27*	GyrA-S83I, ParC-S80I	IncFIB (K),IncF(K), Col440I,Col440II, IncX3	*iutA; fyuA irp2; traT*	*ybt 9;ICEKp3*
KPC-3-producing *K. pneumoniae* (VG313)	January	Lisbon	MEM IPM ERT	147	*bla*_TEM-1A;_ *bla*_SHV-11;_*bla*_KPC-3,_ *sul2;oqxB/A; drfA14*	GyrA-S83I, ParC-S80I	IncFIB, IncFIA	*iutA; fyuA ;irp2*	*ybt 16;ICEKp12*
KPC-3-producing *K. pneumoniae* (VG314)	January	Coimbra	MEM IPM ERT	147	*bla*_TEM-1A;_ *bla*_SHV-11;_*bla*_KPC-3,_ *sul2; oqxB/A; drfA14*	GyrA-S83I, ParC-S80I	IncFIB, IncFIA	*iutA; fyuA;irp2*	*ybt 16*;*ICEKp12*
KPC-3-producing *K. pneumoniae* (VG380)	June	Porto	MEM IPM ERT	392	*aac(6’)-lb-cr, bla*_TEM-1B;_*bla*_SHV-11;_ *bla*_CTX-M-15;_*bla*_KPC-3_*oqxB/A;* *qnrB1*;*tet(A), sul2*	GyrA-S83I, ParC-S80I	IncFIB(K), IncFII(K), IncN	*iutA*	NA^*c*^
OXA-48-producing *E. coli* (VG204)	March	Lisbon		127	*bla*_OXA-48_		NA	*chuA; fyuA;vat; yfcV*	NA

^*a*^ Carbapenem resistance was determined in accordance with EUCAST breakpoint guidelines 2023 (14). Ertapenem resistance MIC >0.5 mg/L; Imipenem resistance MIC >4 mg/L; Meropenem resistance MIC >8 mg/L.

^*b*^ The virulence factors present in this table are the ones associated with uropathogenic lineages. The complete list of virulence factors for strain VG204 is described in Table S9.

^*c*^ NA—not applicable.

The only resistance gene harbored by the carbapenemase-producing *E. coli* was the *bla*_OXA-48_ gene (VG204 strain) (Table 2). MIC determination revealed resistance only to ampicillin (MIC >16 mg/L) and amoxicillin in combination with clavulanic acid (MIC >8/4 mg/L), being fully susceptible to third-generation cephalosporins (MIC ≤1 mg/L) and reduced susceptibility to carbapenems. This *E. coli* strain belonged to the ST127 uropathogenic pathotype (UPEC). In addition to having the virulence factors (VF) encoding genes characteristic of a UPEC (*chuA*, *fyuA*, *vat,* and *yfcV*) (33), it also has other VF-encoding genes which contribute to its virulent profile. The complete list of VF-encoding genes is present in Table S9 (Table S9: Complete list of virulence factors encoding genes found on the OXA-48-producing *E. coli* ST127 strain).

### Phylogenetic analysis

**Fig 1:**
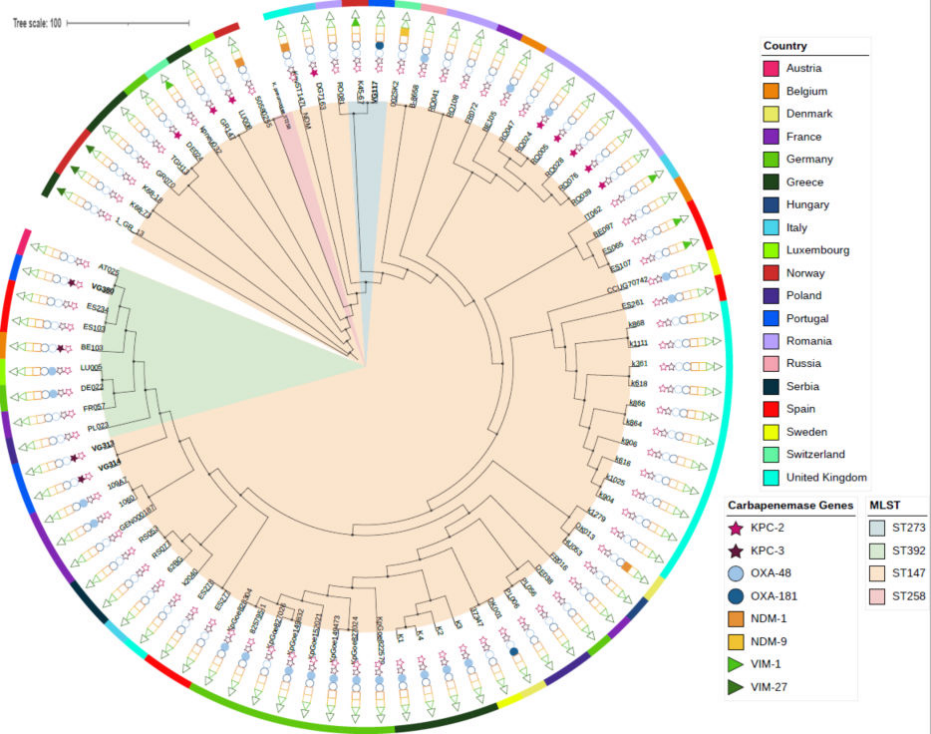
SNP-based phylogenetic tree for *Klebsiella pneumoniae* Clonal Group 147 in Europe from human hosts, characterized according to their carbapenemase genes and country of isolation. Blank symbols represent non-carbapenemase-producing strains. The different clades are colored according to their sequence type. The strains described in this study are in bold.

Phylogenetic analysis showed that our strains are related to other *K. pneumoniae* belonging to the CG 147, previously described in European countries and of human origin. Figure 1 shows that the OXA-181-producing *K. pneumoniae* VG117 strain clusters with a Norwegian VIM-1-producing strain, which might indicate the capacity of the ST273 lineage to acquire different antimicrobial resistance genes. Similarly, the KPC-3-producing *K. pneumoniae* VG380 strain is clustering with strains that do not harbor any carbapenemase gene. This analysis also revealed that KPC-3-producing *K. pneumoniae* ST147 VG313 and VG314 strains belonged to the same clone, with only four single nucleotide polymorphisms (SNPs) difference, which concurs with the breakpoint established of the intra-clade relationship of 10 SNPs difference (34).

No phylogenetic analysis was performed for the OXA-48-producing *E. coli* ST127 strain as it is a commonly described lineage across the globe, both in humans and animals (35).

### Detection of plasmid derived contigs

**Fig 2:**
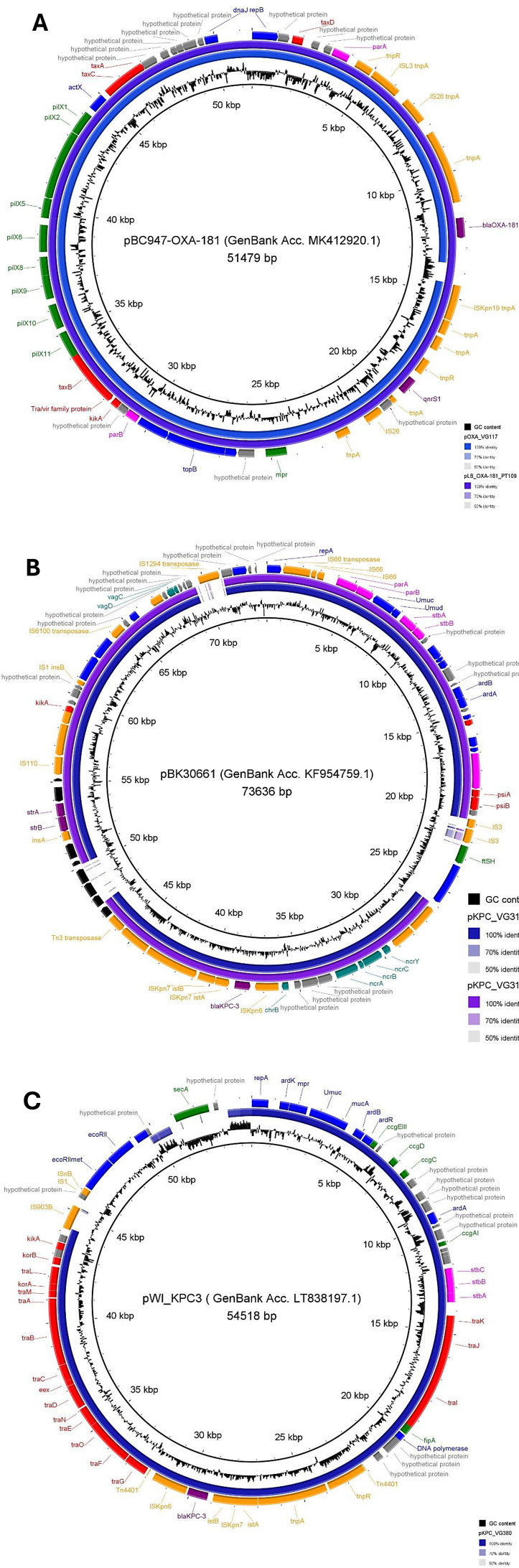
Plasmid alignment comparison between *de novo* assembled plasmids and respective references. (A) pOXA_VG117 (GenBank accession number PRJNA808048) (blue) against pBC947-OXA-181 (GenBank Acc. MK412920.1) and pLB_OXA-181_PT109 (GenBank Acc. CP041033) (purple); (B) pKPC_VG313 and pKPC_VG314 against pBK30661 (GenBank Acc. KF954759.1); (C) – pKPC_VG380 (13) against pWI_KPC3 (GenBank Acc. LT838197.1). Genes are represented by colored blocks: purple, resistance genes; blue, DNA replication, regulation, and restriction systems; red, conjugation-association genes; fuchsia, genes associated with partition and stability systems; orange, transposons, insertion sequences (IS) and transposase genes; teal, genes associated with resistance to heavy metals; black, sulfonamide resistance pathway genes; green, other genes; gray, hypothetical proteins. Image generated using BRIG 0.95, available at http://brig.sourceforge.net/.

The *bla*_OXA-181_ gene was located on insertion sequence IS*Kpn19* on a ~50 kb IncX3-type plasmid (pOXA_VG117), as previously described (12, 36). When using a formerly described IncX3 plasmid of clinical human origin containing the *bla*_OXA-181_ gene as a reference for BLAST (pBC947-OXA-181) (37), several contigs of OXA-181-producing *K. pneumoniae* VG117 strain showed a large extent of homology with the reference plasmid (Fig. 2A). Both resistance genes *bla*_OXA-181_ and *qnrS1* were part of a composite transposon flanked at both ends by IS26. When comparing our plasmid to the previously described pLB_OXA-181_PT109 (GenBank Acc. CP041033), a complete alignment of the plasmids was observed. Plasmid pLB_OXA-181_PT109 was found on a commensal OXA-181-producing *E. coli* strain from a Portuguese dog (12).

For all strains, the *bla*_KPC-3_ gene was located on transposon Tn*4401d—*an isoform of Tn*4401* with 68 bp deletion between ISKpn7 and *bla*_KPC-3_ (38, 39). This isoform has been described in different plasmid types and, similarly to previous reports, VG313 and VG314 strains contained Tn*4401d* in a ~140 kb IncFIA type plasmid (pKPC_VG313 and pKPC_VG314), whereas in VG380, the transposon was located on a ~ 50 kb IncN-type plasmid (pKPC_VG380, pMLST ST15).

A previously described IncFIA plasmid carrying the *bla*_KPC-3_ gene in Tn*4401d* (pBK30661) from a human nosocomial strain (40) was used as a reference for the BLAST analysis. Several contigs for the KPC-3-producing *K. pneumoniae* ST147 VG313 and VG314 strains shared a 100% identical IncFIA plasmid between them, with a high percentage of homology to the reference (Fig. 2B).

For the KPC-3-producing *K. pneumoniae* ST392 VG380 strain, its plasmid-derived contig has been previously described by us in an IncN plasmid from a human nosocomial strain (Fig. 2C) (13).

No plasmid replicons were detected for the OXA-48-producing *E. coli* strain.

## DISCUSSION

In this retrospective study, we found a frequency of 0.51% carbapenem-resistant Enterobacterales strains causing infections in companion animals in Portugal in 2020. Furthermore, we established the horizontal transmission of mobile genetic elements carrying carbapenemase genes in *K. pneumoniae* strains in human and animal settings. Our study is in agreement with the hypothesized high probability for the cross-transmission of resistant bacteria within the One Health context (8, 10, 41).

With the analysis of the antimicrobial susceptibility data, we observed a prevalence of 20.8% for ESBL-producing Enterobacterales among the initial collection of clinical strains. Studies evaluating a heterogeneous collection of Enterobacterales isolates are scarce. In a Swiss study using data from 2012 to 2016 (42), the same prevalence as ours was reported. In another study, only on clinical *E. coli* strains from the UK, 18.7% of the ESBL-producing strains were reported (43). The resemblance observed is of concern since it demonstrates that, despite efforts to decrease the usage of third-generation cephalosporin in veterinary medicine in Europe (44), resistance to critically important antimicrobials for human medicine is still high (30).

In the present study, five CPE strains (0.51%) were found (three KPC-3-producing *K. pneumoniae,* one OXA-181-producing *K. pneumoniae,* and one OXA-48-producing *E. coli* strains). Our result is equivalent to a German study of a large collection of Enterobacterales strains obtained between 2009 and 2016, which reported a prevalence of 0.64% of CPE, all of which were OXA-48-producing strains (7).

To the best of our findings, none of the affected animals received treatment with carbapenems—the oeasophasgotmy tube was removed and surgical cleaning of the wound was performed, followed by antimicrobial treatment with metronidazole, enrofloxacin, and amoxicillin-clavulanic acid. For the dog with an upper respiratory tract infection, treatment with trimethoprim-sulfamethoxazole was effective (13). The dog with a urinary tract infection died before the diagnostic laboratory completed the analysis. In the two other instances, the small animal veterinary practices, where the animals were being monitored, did not reply to our inquiries. Overall, three animals were not treated with carbapenems. Thus, no selective pressure from these antibiotics was exerted. As animals can be reservoirs for MDR bacteria, particularly ESBL and CPE Enterobacterales (41, 45), active surveillance by veterinary diagnostic laboratories is essential.

Of the carbapenemase-producing *K. pneumoniae* strains, only one was identified as a possible CPE by the diagnostic laboratory. This identification was accidental since MIC evaluation was only performed upon the clinician’s request. The evaluation of carbapenem susceptibility in clinical microbiology veterinary diagnostic laboratories is not mandatory since these antibiotics are not for use in veterinary medicine (6). Despite the low frequency observed, summarized in Table 1, our results emphasize the need for implementing detection methods for carbapenemase-producing strains in veterinary medicine (45). The main purpose of screening for carbapenemase-producing strains would be to minimize its spread, which ultimately impacts human health as it challenges the use of carbapenems as a human therapeutic option. Such implementation can be quite easy and affordable to accomplish in the veterinary microbiology routine workflow, as previously described (8). Resistance to carbapenems was observed on all our KPC-3-producing *K. pneumoniae* strains. Yet, they remain susceptible to the combination disc of ceftazidime-avibactam, which is the therapeutic choice for infections caused by carbapenem-resistant strains (46). Furthermore, all of them were ESBL-producing strains, harboring *bla*_CTX-M-15_ and *bla*_SHV-11_ genes. In addition, resistance genes to other antibiotic classes were identified during WGS characterization, which further highlights their MDR profile. MLST analysis also showed that the OXA-181-producing *K. pneumoniae* ST273 strain, KPC-3-producing *K. pneumoniae* strains ST147 strains, and the KPC-3-producing *K. pneumoniae* ST392 strain are all part of the clonal group 147.

The Human hospital setting in Portugal exhibits a high prevalence of *K. pneumoniae* CG147 strains in circulation (5, 47). *K. pneumoniae* ST147 is internationally disseminated with an increasing report of carbapenemase carriage, particularly of *bla*_OXA-48_-like genes (Fig. 1) (31). In Portugal, reports of ST147 *K. pneumoniae* strains harboring the *bla*_OXA-181_ or *bla*_KPC-3_ genes are most frequent in human clinical strains (5, 34, 47). This lineage is classified as a high-risk clone, playing a major role in the spread of resistance (48). Hence, its detection in companion animals is of great public health concern. As for ST392, there are still few reports in Europe—this might be as it was the last lineage to diverge from ST147 (31). The fact that our KPC-3-producing *K. pneumoniae* is closely related to the human Spanish and Austrian strains, despite neither carrying a carbapenemase gene, demonstrates how this ST392 is distributed across Europe. Lastly, the genetic background of the *K. pneumoniae* ST273 lineage has been described in Italy, Russia, Norway, and the United Kingdom, where it was associated with *bla*_KPC-like_, *bla*_VIM-1_, and *bla*_NDM-1_, respectively (49–52). Although the epidemiology of carbapenemase-producing *K. pneumoniae* is changing across Europe (53), ST273 is still found in low numbers. Thus, the association of the ST273 genetic background with different carbapenemase genes highlights the need for continued monitoring (Fig. 1).

Concerning plasmids carrying carbapenemase genes, both KPC-3-producing *K. pneumoniae* ST147 strains harbored the *bla*_KPC-3_ gene on an IncFIIA type plasmid, while KPC-3-producing *K. pneumoniae* ST392 strain harbored the *bla*_KPC-3_ gene on an IncN type plasmid. On these three strains, the *bla*_KPC-3_ gene was located on Tn*4401d*. Portuguese studies on human nosocomial strains have shown that KPC-3-producing *K. pneumoniae* ST147 can carry multiple plasmid replicons, including the IncFIIA and IncN-types, with the *bla*_KPC-3_ gene always located on transposon Tn*4401d* (5, 34, 39, 47). Similarly, our strains harbor the carbapenemase gene in different types of plasmids but in the same genetic environment. These results are worrisome as they might indicate the dissemination/transmission of carbapenemase genes through transposon Tn*4401d* in Veterinary medicine may be fast, thus following the same path as human nosocomial strains.

As for the OXA-181-producing *K. pneumoniae* strain, the *bla*_OXA-181_ gene was located on an IncX3-type plasmid. Moreover, our group had previously described an OXA-181-producing *E. coli* strain from a dog’s feces (12). In both instances, the two strains shared an identical composition of the mobile genetic elements—the transposon where the carbapenemase gene is located, and the plasmid type (Fig. 2A).

Conjugative plasmids have been associated with the horizontal gene transfer of antibiotic resistance genes (54). All carbapenemase-carrying plasmids identified in this study are conjugative plasmids as they have all the characteristic genetic machinery present (55) (Fig. 2). Such results are alarming as they may point to the dissemination and or horizontal transmission of carbapenemase genes in veterinary medicine.

The OXA-48-producing *E. coli* ST127 VG204 strain was a virulent UPEC pathotype, albeit very susceptible to the third-generation cephalosporins, it presented a reduced susceptibility to carbapenems, which underrates its epidemiological value (16, 56). In 2013, an OXA-48-producing *E. coli* ST127 strain was found to have the resistance gene inserted into its chromosome (57). The lack of plasmid replicon identification on our OXA-48-producing *E. coli* strain during WGS analysis may probably mean that the *bla*_OXA-48_ gene is inserted on the chromosome as well.

We recognize that our study has some limitations. First, long-read sequencing needs to be performed to complement the information obtained during the WGS analysis—particularly, to better evaluate the genetic environment surrounding the *bla*_OXA-48_ gene. In addition, an underestimation of carbapenem-resistant strains is possible since we excluded from the study other resistant mechanisms to carbapenems, such as loss/alteration of porins.

This is the first study on the prevalence of carbapenemase-producing Enterobacterales clinical strains from companion animals in Portugal. Although the overall prevalence of CPE in companion animals is quite low, it is important for veterinary diagnostic laboratories to perform phenotypic and/or genotypic carbapenemase detection methods. The similitude observed between the mobile genetic elements carrying carbapenemase genes in veterinary medicine and Human Medicine suggests that circulation in a One Health context is occurring.

Furthermore, infection prevention and control measures should be implemented in small animal veterinary practices to prevent the dissemination of resistance to this high priority critically important antimicrobials in human medicine to the community environment.

## Data Availability

The genomes generated and analyzed during this study are available at the NCBI GenBank under the BioProject Acc. Number PRJNA808048.

## SUPPLEMENTAL MATERIAL

Tables S1 to S9 spectrum.03416-23-s0001.docx Supplemental tables with data characterization and reference sequences used for phylogenetic tree and plasmid reconstruction.

Supplemental File 10 spectrum.03416-23-s0002.xlsx SNP matrix used for phylogenetic tree generation of *K. pneumoniae* strains.

ASM does not own the copyrights to Supplemental Material that may be linked to, or accessed through, an article. The authors have granted ASM a non-exclusive, world-wide license to publish the Supplemental Material files. Please contact the corresponding author directly for reuse.
