# Supplementary material for: Carbapenemase-producing Enterobacterales strains causing infections in companion animals—Portugal
Source: Microbiol Spectr. 2024 Mar 6;12(4):e03416-23. doi: 10.1128/spectrum.03416-23 (PMC10986603; doi:10.1128/spectrum.03416-23)
Supplement: Tables S1 to S9 — Supplemental tables with data characterization and reference sequences used for phylogenetic tree and plasmid reconstruction. [file spectrum.03416-23-s0001.docx]

| **Antibiotic Tested** | **Percentage of susceptible clinical strains** | **Percentage of susceptible to**  **increase exposure clinical strains** | **Percentage of resistant**  **clinical strains** |
| --- | --- | --- | --- |
| Amikacin (30µg) | 94.9 | 3.6 | 1.0 |
| Ampicillin (10µg) | 36.3 | 8.9 | 54.4 |
| Amoxicillin in association with clavulanic acid (30µg) | 65.5 | 8.4 | 25.3 |
| Cefoxitin (30µg) | 80.6 | 4.2 | 14.5 |
| Cephalothin (30µg) | 36.1 | 20.2 | 43.4 |
| Cefotaxime (30µg) | 73.2 | 3.7 | 22.7 |
| Ceftazidime (30µg) | 81.3 | 3.6 | 14.4 |
| Enrofloxacin (5µg) | 64.2 | 7.6 | 27.9 |
| Gentamicin (10µg) | 83.2 | 1.8 | 14.5 |
| Tetracycline (30µg) | 36.1 | 5.9 | 57.4 |
| Trimethoprim/sulfamethoxazole (25µg) | 62.0 | 1.6 | 35.8 |

**Supplementary Table 1**. Antimicrobial susceptibility results of the clinical Enterobacterales strains isolated from companion animals (n=977)

Enterobacterales strains include *E. coli*, *Klebsiella spp*., *Enterobacter spp*., *Citrobacter spp*., *Serratia spp*. and *Proteus spp*.

**Supplementary Table 2**. Data used to generate *Klebsiella pneumoniae* Clonal Group 147 phylogenetic tree. All *K. pneumoniae* strains are of human origin.

| **Strain Identification** | **GenBank Accession Number** | **Country** | **ST** | **Infection Type** | **Carbapenemase gene** |
| --- | --- | --- | --- | --- | --- |
| K45-67 | GCA_001483725.1 | Norway | 273 | NA | *bla*_VIM-1_ |
| DE022 | GCA_900512335.1 | Germany | 392 | infection (NS) | *bla*_OXA-48_ |
| LU005 | GCA_900503635.1 | Luxembourg | 392 | infection (NS) | *bla*_OXA-48_ |
| BE103 | GCA_900507095.1 | Belgium | 392 | infection (NS) | *bla*_KPC-3_ |
| AT025 | GCA_900508275.1 | Austria | 392 | infection (NS) | NA |
| ES103 | GCA_900516645.1 | Spain | 392 | infection (NS) | NA |
| ES234 | GCA_900517365.1 | Spain | 392 | infection (NS) | NA |
| FR057 | GCA_900511745.1 | France | 392 | infection (NS) | NA |
| PL023 | GCA_900511445.1 | Poland | 392 | infection (NS) | NA |
| 002SK2 | GCA_002848605.1 | Switzerland | 147 | NA | *bla*_NDM-9_ |
| B-8658 | GCA_004310675.1 | Russia | 147 | NA | *bla*_OXA-48_ |
| LU008 | GCA_900503645.1 | Luxembourg | 147 | infection (NS) | *bla*_KPC-2_ |
| GR147 | GCA_900502395.1 | Greece | 147 | infection (NS) | *bla*_KPC-2_ |
| 1_GR_13 | GCA_001701425.2 | Greece | 147 | NA | *bla*_VIM-27_ |
| K68-18 | GCA_001462825.1 | Norway | 147 | NA | *bla*_VIM-27_ |
| K68-73 | GCA_001462845.1 | Norway | 147 | NA | *bla*_VIM-27_ |
| kpneu032 | GCA_900608075.1 | Switzerland | 147 | infection (NS) | *bla*_VIM-1_ |
| TGH13 | GCA_001746535.1 | Greece | 147 | NA | NA |
| GR070 | GCA_900500845.1 | Greece | 147 | infection (NS) | NA |
| DE024 | GCA_900512345.2 | Germany | 147 | NA | *bla*_KPC-2_ |
| RO005 | GCA_900504855.1 | Romania | 147 | infection (NS) | *bla*_KPC-2_ |
| **Strain Identification** | **GenBank Accession Number** | **Country** | **ST** | **Infection Type** | **Carbapenemasegene** |
| RO028 | GCA_900504585.1 | Romania | 147 | NA | *bla*_KPC-2_ |
| RO039 | GCA_900504685.1 | Romania | 147 | NA | *bla*_KPC-2_ |
| RO076 | GCA_900504225.1 | Romania | 147 | infection (NS) | *bla*_KPC-2_ |
| DG7163 | GCA_003227055.1 | Italy | 147 | colonization | *bla*_KPC-2_ |
| ES065 | GCA_900516415.1 | Spain | 147 | infection (NS) | *bla*_VIM-1_ |
| ES107 | GCA_900501685.1 | Spain | 147 | infection (NS) | *bla*_VIM-1_ |
| IT062 | GCA_900513215.1 | Italy | 147 | infection (NS) | *bla*_VIM-1_ |
| RS053 | GCA_900505345.1 | Serbia | 147 | infection (NS) | NA |
| RS077 | GCA_900505425.1 | Serbia | 147 | infection (NS) | NA |
| ES261 | GCA_900517095.1 | Spain | 147 | infection (NS) | *bla*_OXA-48_ |
| ES261 | GCA_900517095.1 | Spain | 147 | infection (NS) | *bla*_OXA-48_ |
| RO047 | GCA_900504015.1 | Romania | 147 | infection (NS) | NA |
| BE097 | GCA_900507055.1 | Belgium | 147 | infection (NS) | NA |
| FR016 | GCA_900510435.1 | France | 147 | infection (NS) | NA |
| HU053 | GCA_900517045.1 | Hungary | 147 | infection (NS) | NA |
| 62BG | GCA_000822405.1 | Italy | 147 | NA | NA |
| k1025 | GCA_900085785.1 | United Kingdom | 147 | bacteraemia | NA |
| k1111 | GCA_900086215.1 | United Kingdom | 147 | infection (NS) | NA |
| k1279 | GCA_900084235.1 | United Kingdom | 147 | infection (NS) | NA |
| k2040 | GCA_900086515.1 | United Kingdom | 147 | infection (NS) | NA |
| k361 | GCA_900086065.1 | United Kingdom | 147 | infection (NS) | NA |
| k616 | GCA_900084905.1 | United Kingdom | 147 | infection (NS) | NA |
| k864 | GCA_900085105.1 | United Kingdom | 147 | infection (NS) | NA |
| k866 | GCA_900085095.1 | United Kingdom | 147 | infection (NS) | NA |
| **Strain Identification** | **GenBank Accession Number** | **Country** | **ST** | **Infection Type** | **Carbapenemasegene** |
| k868 | GCA_900085115.1 | United Kingdom | 147 | infection (NS) | NA |
| k906 | GCA_900085145.1 | United Kingdom | 147 | infection (NS) | NA |
| k618 | GCA_900086085.1 | United Kingdom | 147 | infection (NS) | NA |
| k904 | GCA_900086155.1 | United Kingdom | 147 | infection (NS) | NA |
| RO081 | GCA_900504315.1 | Romania | 147 | infection (NS) | NA |
| ES277 | GCA_900517665.1 | Spain | 147 | infection (NS) | NA |
| ES278 | GCA_900517655.1 | Spain | 147 | infection (NS) | NA |
| GEN000187 | GCA_004145685.1 | France | 147 | NA | NA |
| DK013 | GCA_900502585.1 | Denmark | 147 | infection (NS) | *bla*_NDM-1_ |
| BE105 | GCA_900507565.1 | Belgium | 147 | infection (NS) | *bla*_OXA-48_ |
| RO024 | GCA_900504755.1 | Romania | 147 | infection (NS) | *bla*_OXA-48;_ *bla*_KPC-2_ |
| 1060 | GCA_004005735.1 | France | 147 | NA | *bla*_OXA-48_ |
| 109A7 | GCA_004005185.1 | France | 147 | NA | *bla*_OXA-48_ |
| DE038 | GCA_900512445.1 | Germany | 147 | infection (NS) | NA |
| PL006 | GCA_900511365.1 | Poland | 147 | colonization | NA |
| PL056 | GCA_900511505.1 | Poland | 147 | infection (NS) | NA |
| PL056 | GCA_900511505.1 | Poland | 147 | infection (NS) | NA |
| 37347 | GCA_003856595.1 | Sweden | 147 | colonization | NA |
| DK001 | GCA_900502525.1 | Denmark | 147 | infection (NS) | *bla*_OXA-181_ |
| K1 | GCA_003034565.1 | Greece | 147 | bacteraemia | *bla*_OXA-48_ |
| K2 | GCA_003034485.1 | Greece | 147 | bacteraemia | *bla*_OXA-48_ |
| K3 | GCA_003034435.1 | Greece | 147 | bacteraemia | *bla*_OXA-48_ |
| K4 | GCA_003034385.1 | Greece | 147 | bacteraemia | *bla*_OXA-48_ |
| 825795-1 | GCA_001956965.1 | Germany | 147 | colonization | *bla*_OXA-48_ |
| **Strain Identification** | **GenBank Accession Number** | **Country** | **ST** | **Infection Type** | **Carbapenemase_gene** |
| KpGoe149473 | GCA_001908595.1 | Germany | 147 | pneumonia | *bla*_OXA-48_ |
| KpGoe149832 | GCA_001908875.1 | Germany | 147 | peritonitis | *bla*_OXA-48_ |
| KpGoe828304 | GCA_001908695.1 | Germany | 147 | colonization | *bla*_OXA-48_ |
| KpGoe152021 | GCA_001908675.1 | Germany | 147 | peritonitis | *bla*_OXA-48_ |

NA – Not applicable; NS – Not Specified

**Supplementary Table 3**. Accession numbers and relevant information on plasmids used for the study’s contigs circularization

| Plasmid Identification | GenBank Accession Number | Plasmid Replicon | Carbapenemase Gene | Country | Origin |
| --- | --- | --- | --- | --- | --- |
| pBC947-OXA-181 | MK412920.1 | IncX3 | *bla*_OXA-181_ | United Arab Emirates | Nosocomial Infection - bacteremia |
| pBK30661 | KF954759.1 | IncFIA | *bla*_KPC-3_ | United States | Nosocomial Infection – urinary tract infection |
| pWI_KPC3 | LT838197.1 | IncN | *bla*_KPC-3_ | France | Nosocomial Infection - Not specified |

**Supplementary Table 4**. Routine carbapenem minimal inhibitory concentration results for clinical strains (n=62)

| **Strains (n=62)** | **MIC^a^ range for Imipenem** | **Number of strains resistant to Imipenem** | **MIC^a^ range for Meropenem** | **Number of strains resistant to Meropenem** | **MIC^a^ range for Ertapenem** | **Number of strains resistant to Ertapenem** |
| --- | --- | --- | --- | --- | --- | --- |
| *Klebsiella* spp. (n=19) | ≤1- 4 mg/L | 0 | ≤1 | 0 | ≤0,5- >1 mg/L | 1 |
| *Proteus* spp. (n=15) | ≤1- >8 mg/L | 2^b^ | ≤1- >4 mg/L | 0 | ≤0,5- >1 mg/L | 2 |
| *E*. *coli* (n=13) | ≤1mg/L | 0 | ≤1 mg/L | 0 | ≤0,5 mg/L | 0 |
| *Serratia* spp. (n=5) | ≤1mg/L | 0 | ≤1 mg/L | 0 | ≤0,5 mg/L | 0 |
| *Enterobacter* spp. (n=10) | ≤1mg/L | 0 | ≤1 mg/L | 0 | ≤0,5- 1 mg/L | 0 |

^a^Carbapenem resistance was determined in accordance to EUCAST breakpoint guidelines 2023 (13). Ertapenem resistance MIC >0.5 mg/L; Imipenem resistance MIC > 4 mg/L; Meropenem resistance MIC >8 mg/L.

^b^*Morganellaceae*. are intrinsically resistant to low concentrations of imipenem, thus requiring exposure to high doses of imipenem (13).

**Supplementary Table 5**. Distribution of strains amongst the study groups, according to the animal species and type of infection

|  | **Clinical strains with an ESBL phenotype**  **(n = 204)** | **Clinical strains with a possible OXA-48-like phenotype (n = 34)** | **Clinical strains with an MDR profile (n = 23)** | **Total** |
| --- | --- | --- | --- | --- |
| **Animal Species (%)** |  |  |  |  |
| Dog | 64.7% (n =132) | 64.7 % (n =22) | 65.2% (n =15) | 169 |
| Cat | 28.9% (n =59) | 26.5 % (n =9) | 26.1% (n =6) | 74 |
| Other | 6.4 % (n =13) | 8.8% (n =3) | 8.7% (n =2) | 18 |
| **Infection Type (%)** | a | b |  |  |
| UTI | 42.1 % (n =86) | 38.2% (n =13) | 60.9% (n =14) | 113 |
| SSTI | 34.3 % (n =70) | 50.0 % (n =17) | 13.0% (n =3) | 90 |
| URTI | 13.2 % (n =27) | 2.9% (n =1) | 8.7% (n =2) | 30 |
| Otitis externa | 9.8 % (n =20) | 5.9% (n =2) | 17.4% (n =4) | 26 |

ESBL – Extended Spectrum β-Lactamases; MDR – Multidrug Resistant; SSTI – Skin and soft tissue infections; URTI- Upper respiratory tract infection; UTI – Urinary tract infection.

^a^ Infection characterized as SSTI/UTI (n =1); ^b^ Haemoculture (n =1)

**Supplementary Table 6**. β-lactamase genes detected in clinical strains from companion animal (n=261)

| **β-lactamases genes detected** | **Clinical strains with an ESBL phenotype** | **Clinical strains with a possible OXA-48-like phenotype** | **Clinical strains with an MDR profile** |
| --- | --- | --- | --- |
| *bla*_TEM-1_ | *K. pneumoniae* (n=49)  *E. coli* (n=34)  *E. cloacae* complex (n=23)  *P. mirabilis* (n=10)  *P. vulgaris* (n=5)  *K. variicola* (n=2)  *M. morganni* (n=2)  *K. quasipneumoniae* (n=1) | *E. coli* (n=7)  *P. mirabilis* (n=1) | *E. coli* (n=5)  *P. mirabilis* (n=8)  *P. vulgaris* (n=2)  *K. pneumoniae* (n=1)  *K. quasipneumoniae* (n=1) |
| *bla*_TEM-135_ | *E. coli* (n=1) |  | *E. coli* (n=1) |
| *bla*_TEM-156_ | *P. mirabilis* (n=1) |  |  |
| *bla*_TEM-32_ |  |  | *E. coli* (n=1) |
| *bla*_TEM-35_ |  | *E. coli* (n=1) |  |
| *bla*_SHV-28_ | *K. pneumoniae* (n=6)  *E. cloacae* complex (n=1) |  | *K. pneumoniae* (n=1) |
| *bla*_SHV-11_ | *K. pneumoniae* (n=4) |  |  |
| *bla*_SHV-12_ | *E. cloacae* complex (n=3)  *E. coli* (n=3)  *K. quasipneumoniae* (n=1) |  |  |
| *bla*_SHV-1_ | *K. pneumoniae* (n=1) |  |  |
| *bla*_SHV-76_ | *K. pneumoniae* (n=1) |  |  |
| *bla*_SHV-2_ | *K. pneumoniae* (n=1) |  |  |
| **β-lactamases genes detected** | **Clinical strains with an ESBL phenotype** | **Clinical strains with a possible OXA-48-like phenotype** | **Clinical strains with an MDR profile** |
| *bla*_CTX-M-15_ | *K. pneumoniae* (n=48)  *E. coli* (n=23)  *E. cloacae* complex (n=21) *K. variicola* (n=3)  *P. mirabilis* (n=3)  *M. morganni* (n=2)  *K. quasipneumoniae* (n=1) *P. vulgaris* (n=1) |  |  |
| *bla*_CTX-M-1_ | *E. coli* (n=8)  *K. pneumoniae* (n=2)  *E. cloacae* complex (n=1)  *P. mirabilis* (n=1) |  |  |
| *bla*_CTX-M-14_ | *P. mirabilis* (n=5)  *E. coli* (n=4)  *P. vulgaris* (n=3)  *E. cloacae* complex (n=1)  *K. quasipneumoniae* (n=1) |  |  |
| *bla*_CTX-M-65_ | *E. coli* (n=6)  *P. vulgaris* (n=1) |  |  |
| *bla*_CTX-M-9_ | *E. cloacae* complex (n=1)  *K. quasipneumoniae* (n=1) |  |  |
| *bla*_CTX-M-55_ | *E. coli* (n=6) |  |  |
| *bla*_CTX-M-156_ | *E. cloacae* complex (n=1) |  |  |
| *bla*_CTX-M-28_ | *E. coli* (n=1) |  |  |
| *bla*_CTX-M-3_ | *K. pneumoniae* (n=1) |  |  |
| **β-lactamases genes detected** | **Clinical strains with an ESBL phenotype** | **Clinical strains with a possible OXA-48-like phenotype** | **Clinical strains with an MDR profile** |
| *bla*_CTX-M-32_ | *E. coli* (n=1) |  |  |
| *bla*_CMY-2_ | *E. coli* (n=10)  *P. mirabilis* (n=3)  *P. vulgaris* (n=2)  *E. cloacae* complex (n=1)  *K. pneumoniae* (n=2)  *C. murliniae* (n=1) |  |  |
| *bla*_DHA-1_ | *K. pneumoniae* (n=18)  *P. mirabilis* (n=3)  *E. cloacae* complex (n=2)  *E. coli* (n=1)  *M. morganni* (n=1) | *E. coli* (n=1) |  |
| *bla*_DHA-16_ | *K. pneumoniae* (n=1) |  |  |
| *bla*_DHA-17_ | *M. morganni* (n=1) | *P. mirabilis* (n=1) |  |
| *bla*_DHA-5_ | *M. morganni* (n=2)  *E. coli* (n=1) |  |  |
| *bla*_MIR-like_ | *E. cloacae* complex (n=1)  *K. pneumoniae* (n=1) |  |  |

ESBL – Extended Spectrum β-Lactamases; MDR – Multidrug Resistant

**Supplementary Table 7.** Assemblies properties following WGS analysis

| **Strains tested** | **N50** | **Total Assembly Length** | **Reference Length** | **GC Content (%)** | **GC Content**  **Reference (%)** | **Nº of contigs** | **L50** | **Depth Coverage** |
| --- | --- | --- | --- | --- | --- | --- | --- | --- |
| OXA-181-producing  *Klebsiella pneumoniae* (VG117) | 166995 | 5669435 | 5279178 (GenBank Acc. CP102077.1) | 57.12 | 57.17 | 340 | 13 | 105x (84-119x) |
| OXA-48-producing *Escherichia coli* (VG204) | 159909 | 5347507 | 5184627 (GenBank Acc. HG994856.1) | 50.44 | 50.43 | 1233 | 10 | 105x (77-119x) |
| KPC-3-producing  *Klebsiella pneumoniae*  (VG313) | 278948 | 5544925 | 5438016 (GenBank Acc. CP023839.1) | 57.08 | 57.03 | 151 | 6 | 250x (216-270x) |
| KPC-3-producing  *Klebsiella pneumoniae* (VG314) | 323447 | 5553936 | 5438016 (GenBank Acc. CP023839.1) | 57.08 | 57.03 | 185 | 6 | 250x (231-294x) |
| KPC-3-producing  *Klebsiella pneumoniae* (VG380) | 168573 | 5625432 | 5325495 (GenBank Acc. CP08370.1) | 57.18 | 57.24 | 319 | 11 | 98x (84-108x) |

**Supplementary Table 8**. Antimicrobial MICs and resistance genes identified during WGS analysis on carbapenemase-producing *Klebsiella pneumoniae* strains

| **Strain** | **Antimicrobials Tested** | **MIC mg/L** | **Susceptibility Phenotype^§^** | **AMR genes** |
| --- | --- | --- | --- | --- |
| OXA-181 *K. pneumoniae* VG117 ST273 | Amikacin | ≤8 | S | *aac(6’)-lb-cr* |
|  | Ampicillin | >16 | R | *bla*_SHV-1;_ *bla*_CTX-M-15;_ *bla*_OXA-181_ |
|  | Amoxicillin- Clavulanic Acid | >16/8 | R^†^ | *bla*_CTX-M-15;_ *bla*_OXA-181_ |
|  | Aztreonam | >16 | R | *bla*_CTX-M-15;_ *bla*_OXA-181_ |
|  | Cefotaxime | >32 | R | *bla*_CTX-M-15;_ *bla*_OXA-181_ |
|  | Ceftazidime | >16 | R | *bla*_CTX-M-15;_ *bla*_OXA-181_ |
|  | Ciprofloxacin | >2 | R | *OqxB/A; qnrS1;* GyrA-S83I; ParC-S80I |
|  | Colistin | ≤2 | S | NA |
|  | Ertapenem | >1 | R | *bla*_OXA-181_ |
|  | Gentamicin | ≤2 | S | NA |
|  | Imipenem | ≤1 | S | *bla*_OXA-181_ |
|  | Meropenem | ≤1 | S | *bla*_OXA-181_ |
|  | Tetracycline | >8 | R^†^ | *tetD* |
|  | Trimethoprim/Sulfamethoxazole | >4/76 | R^†^ | *sul1; OqxB/A; dfrA27* |
| KPC-3 *K. pneumoniae* VG313 ST147 | Amikacin | ≤8 | S | NA |
|  | Ampicillin | >16 | R | *bla*_TEM-1A;_ *bla*_SHV-11;_ *bla*_KPC-3_ |
|  | Amoxicillin- Clavulanic Acid | >16/8 | R^†^ | *bla*_KPC-3_ |
|  | Aztreonam | >16 | R | *bla*_KPC-3_ |
|  | Cefotaxime | >32 | R | *bla*_KPC-3_ |
| **Strain** | **Antimicrobials Tested** | **MIC mg/L** | **Susceptibility Phenotype^§^** | **AMR genes** |
| KPC-3 *K. pneumoniae* VG313 ST147 | Ceftazidime | >16 | R | *bla*_KPC-3_ |
|  | Ciprofloxacin | >2 | R | GyrA-S83I; ParC-S80I |
|  | Colistin | ≤2 | S | NA |
|  | Ertapenem | >1 | R | *bla*_KPC-3_ |
|  | Gentamicin | ≤2 | S | NA |
|  | Imipenem | >8 | R | *bla*_KPC-3_ |
|  | Meropenem | >8 | R | *bla*_KPC-3_ |
|  | Tetracycline | ≤4 | S^†^ | NA |
|  | Trimethoprim/Sulfamethoxazole | >4/76 | R^†^ | *sul2; OqxB/A; drfA14* |
| KPC-3 *K. pneumoniae* VG314 ST147 | Amikacin | ≤8 | S | NA |
|  | Ampicillin | >16 | R | *bla*_TEM-1A;_ *bla*_SHV-11;_ *bla*_KPC-3_ |
|  | Amoxicillin- Clavulanic Acid | >16/8 | R^†^ | *bla*_KPC-3_ |
|  | Aztreonam | >16 | R | *bla*_KPC-3_ |
|  | Cefotaxime | >32 | R | *bla*_KPC-3_ |
|  | Ceftazidime | >16 | R | *bla*_KPC-3_ |
|  | Ciprofloxacin | >2 | R | GyrA-S83I; ParC-S80I |
|  | Colistin | ≤2 | S | NA |
|  | Ertapenem | >1 | R | *bla*_KPC-3_ |
|  | Gentamicin | ≤2 | S | NA |
|  | Imipenem | >8 | R | *bla*_KPC-3_ |
|  | Meropenem | >8 | R | *bla*_KPC-3_ |
|  | Tetracycline | ≤4 | S^†^ | NA |
| **Strain** | **Antimicrobials Tested** | **MIC mg/L** | **Susceptibility Phenotype^§^** | **AMR genes** |
|  | Trimethoprim/Sulfamethoxazole | >4/76 | R^†^ | *sul2; OqxB/A; drfA14* |
| KPC-3-*K. pneumoniae* VG380 ST392 | Amikacin | ≤8 | S | *aac(6’)-lb-cr* |
|  | Ampicillin | >16 | R | *bla*_TEM-1B;_ *bla*_SHV-11;_ *bla*_CTX-M-15;_ *bla*_KPC-3_ |
|  | Amoxicillin- Clavulanic Acid | >16/8 | R^†^ | *bla*_CTX-M-15;_ *bla*_KPC-3_ |
|  | Aztreonam | >16 | R | *bla*_CTX-M-15;_ *bla*_KPC-3_ |
|  | Cefotaxime | >32 | R | *bla*_CTX-M-15;_ *bla*_KPC-3_ |
|  | Ceftazidime | >16 | R | *bla*_CTX-M-15;_ *bla*_KPC-3_ |
|  | Ciprofloxacin | >2 | R | *aac(6’)-lb-cr ; OqxB/A; qnrB1*;  GyrA-S83I; ParC-S80I |
|  | Colistin | ≤2 | S | NA |
|  | Ertapenem | >1 | R | *bla*_KPC-3_ |
|  | Gentamicin | ≤2 | S | *aac(6’)-lb-cr* |
|  | Imipenem | >8 | R | *bla*_KPC-3_ |
|  | Meropenem | >8 | R | *bla*_KPC-3_ |
|  | Tetracycline | >8 | R^†^ | *tet(A)* |
|  | Trimethoprim/Sulfamethoxazole | ≤2/73 | S^†^ | *sul2; OqxB/A* |

S – Susceptible; R – Resistant; NA – Not applicable

^§^ Susceptibility phenotype was determined according to EUCAST breakpoint guidelines (13)

^†^ Susceptibility phenotype was determined according to CLSI guidelines (16)

**Supplementary Table 9**. Complete list of virulence factors encoding genes found on the OXA-48-producing *E. coli* ST127 strain*

| **Encoding Gene** | **Virulence Factor** |
| --- | --- |
| *afaD* | Afimbrial Adhesion |
| *astA* | EAST-1 heat stable toxin |
| *chuA* | **Outer membrane hemin receptor** |
| *clbB* | Hybrid non-ribosomal peptide /polyketidemegasynthase |
| *cnf1* | Cytotoxic necrotizing factor |
| *fyuA* | **Siderophore receptor** |
| *gad* | Glutamate decarboxylase |
| *hra* | Heat resistant agglutinin |
| *iroN* | Enterobactin siderophore receptor protein |
| *irp2* | Non-ribosomal peptide synthetase |
| *iss* | Increased serum survival |
| *kpsE* | Capsule polysaccharide export inner membrane protein |
| *kpsMII* | Polysialic acid transport protein |
| *mcmA* | Microcin M |
| *ompT* | Outer membrane protease |
| *papA_F48* | Major pilin subunit F48 |
| *papC* | Outer membrane usher P |
| *sfaD/ sfaE* | S fimbrial/F1C minor subunit |
| *sfaS* | S-fimbriae minor subunit |
| *sitA* | Iron transport protein |
| *tcpC* | Tir domain containing protein |
| *terC* | Tellurium ion resistance protein |
| *usp* | Uropathogenic specific protein |
| *vat* | **Vacuolatin autotransporter toxin** |
| *yfcV* | **Fimbrial protein** |

* Bold indicates the VF-encoding genes associated to uropathogenic lineages.
